# Supplementary material for: The Effect of Higher Level Computerized Clinical Decision Support Systems on Oncology Care: A Systematic Review
Source: Cancers (Basel). 2020 Apr 22;12(4):1032. doi: 10.3390/cancers12041032 (PMC7226340; doi:10.3390/cancers12041032)
Supplement: Supplementary file 1 [file cancers-12-01032-s001.pdf]

Supplementary Materials: The effect of higher level computerized clinical decision support systems on oncology care: a systematic review

Sosse E. Klarenbeek [1,*,†], Harm H.A. Weekenstroo [1,*,†], J.P. Michiel Sedelaar [2], Jurgen J. Fütterer [1], Mathias Prokop [1] and Marcia Tummers [3]

Table S1. Search string.

#	Database	Search terms
1	Pubmed	"Decision Support Systems, Management"[Mesh] OR "Decision Making, Computer-Assisted"[Mesh:NoExp] OR "Diagnosis, Computer-Assisted"[Mesh:NoExp] OR "Therapy, Computer-Assisted"[Mesh:NoExp] OR "Decision Support Systems, Clinical"[Mesh] **OR** ((electronic*[tiab] OR computer*[tiab]) AND (clinical decision support*[tiab] OR CDS[tiab] OR CDSS[tiab] OR decision making[tiab] OR decision aid*[tiab] OR decision support*[tiab] OR disease management[tiab])) **AND** ("Neoplasms"[Mesh] OR "Medical Oncology"[Mesh] OR Neoplasm*[Title/Abstract] OR Cancer*[Title/Abstract] OR Malignan*[Title/Abstract] OR Tumor*[Title/Abstract] OR tumour*[tiab] OR Oncolog*[tiab])<br><br>Time range: 01-01-2000-31-12-2019
2	Embase	1. exp decision support system/<br>2. ((electronic* OR computer*) AND (clinical decision support* OR CDS OR CDSS OR decision making OR decision aid* OR decision support* OR disease management)).ti,ab,kw.<br>3. 1 or 2<br>4. exp neoplasm/<br>5. (medical oncolog* OR neoplasm* OR cancer* OR malignan* OR tumor* OR tumour* OR oncolog*).ti,ab,kw.<br>6. 4 or 5<br>7. 3 and 6<br>Limit 7 to yr=''2000- Current''
3	Cochrane Library	1. MeSH descriptor: [Decision making, computer-assisted] explode all trees<br>2. MeSH descriptor: [Decision support systems, management] this term only<br>3. MeSH descriptor: [Diagnosis, computer-assisted] this term only<br>4. MeSH descriptor: [Therapy, computer-assisted] this term only<br>5. MeSH descriptor: [Decision support systems, clinical] explode all trees<br>6. (((electronic* OR computer*) AND (clinical decision support* OR CDS OR CDSS OR decision making OR decision aid* OR decision support* OR disease management))):ti,ab,kw<br>7. #1 or #2 or #3 or #4 or #5<br>8. MeSH descriptor: [Neoplasms] explode all trees<br>9. ((medical oncolog* OR neoplasm* OR cancer* OR malignan* OR tumor* OR tumour* OR oncolog*)):.ti,ab,kw<br>10. #7 or #8<br>11. #6 and #9<br>With Cochrane Library publication date from Jan 2000 to Dec 2019
4	Web of Science	((electronic* OR computer*) AND ("clinical decision support*" OR CDS OR CDSS OR "decision making" OR "decision aid*" OR "decision support*" OR "disease management"))<br>AND<br>("medical oncolog*" OR neoplasm* OR cancer* OR malignan* OR tumor* OR tumour* OR oncolog*)
5	IEEE	((electronic* OR computer) AND (clinical decision support OR CDS OR CDSS OR decision making OR decision aid OR decision support OR disease management))<br>AND<br>(neoplasm OR cancer OR malignan* OR tumor OR tumour OR oncology)
6	ACM digital library	(electronic* computer) +("clinical decision support" CDS CDSS "decision making" "decision aid" "decision support" "disease management") +(neoplasm cancer malignan* tumor tumour oncology)
